# Mucosal microbiota of intestinal polyps reveals putative biomarkers of colorectal cancer

**DOI:** 10.1038/s41598-018-32413-2

**Published:** 2018-09-18

**Authors:** Marta Mangifesta, Leonardo Mancabelli, Christian Milani, Federica Gaiani, Nicola de’Angelis, Gian Luigi de’Angelis, Douwe van Sinderen, Marco Ventura, Francesca Turroni

**Affiliations:** 1 Laboratory of Probiogenomics, Department of Chemistry, Life Sciences and Environmental Sustainability, University of Parma, Parma, Italy; 2 Gastroenterology and Endoscopy Unit, University Hospital of Parma, Parma, Italy; 3 Department of HPB Surgery and Liver Transplantation, Henri-Mondor Hospital, Université Paris Est-UPEC, Créteil, France; 4 School of Microbiology & APC Microbiome Institute, University College Cork, Cork, Ireland; 5 Microbiome Research Hub, University of Parma, Parma, Italy

## Abstract

The human intestine retains a complex microbial ecosystem, which performs crucial functions that impact on host health. Several studies have indicated that intestinal dysbiosis may impact on the establishment of life-threatening intestinal diseases such as colorectal cancer. An adenomatous polyp is the result of abnormal tissue growth, which is benign but is considered to be associated with a high risk of developing colorectal cancer, based on its grade of dysplasia. Development of diagnostic tools that are based on surveying the gut microbiota and are aimed at early detection of colorectal cancer represent highly desirable target. For this purpose, we performed a pilot study in which we applied a metataxonomic analysis based on 16S rRNA gene sequencing approach to unveil the composition of microbial communities of intestinal polyps. Moreover, we performed a meta-analysis involving the reconstructed microbiota composition of adenomatous polyps and publicly available metagenomics datasets of colorectal cancer. These analyses allowed the identification of microbial taxa such as *Faecalibacterium*, *Bacteroides* and *Romboutsia*, which appear to be depleted in cancerogenic mucosa as well as in adenomatous polyps, thus representing novel microbial biomarkers associated with early tumor formation. Furthermore, an absolute quantification of *Fusubacterium nucleatum* in polyps further compounded the important role of this microorganism as a valuable putative microbial biomarker for early diagnosis of colorectal cancer.

## Introduction

Adenomatous polyps or adenomas have long been recognized as precursors of colorectal cancer^1,2^. Colorectal cancer (CRC) is one of the most commonly diagnosed cancers worldwide and consequently one of the major causes of death in developed countries^3^. It is known that adenomatous polyps in some cases may evolve to colorectal cancer, even though no currently available scientific evidence unequivocally demonstrate in how many years and what are the precise causes of degeneration. So far there is a growing number of studies that highlight a direct correlation between polyp size, histology and progression of this pathology to CRC^4^. Furthermore, the molecular causes promoting such malignant transformation of polyps are essentially unknown^5^. Several factors have been investigated for polyp involvement in cancer development, such as genetics, epigenetics, diet, life style, obesity, alcohol intake and smoking^6^.

In recent decades, it has become evident that gut bacteria and their metabolites may participate in triggering or progression of colorectal cancer through various proposed mechanisms, including the production of reactive oxygen radicals and other genotoxins^7–10^, phenolic compound, and indole production^11^, as well as conversion of dietary factors into carcinogens and tumor promoters^12^, and induction of proinflammatory and procarcinogenic pathways in host epithelial cells^13–15^. These proposed mechanisms have an impact in altering the metabolic environment of the host, which may directly or indirectly influence mutagenesis rates and thus carcinogenesis^16^. Various studies have explored the gut microbiota of individuals with CRC, resulting in the identification of a range of different bacterial groups being associated with carcinogenesis, including *Helicobacter pylori*, *Bacteroides fragilis*, *Fusobacterium nucleatum*, various pathogenic *Escherichia coli*, *Streptococcus gallolyticus* and members of the genus *Clostridium*^17–26^. Although screening and lifestyle play important roles in early detection and prevention of colorectal cancer, the identification of new diagnostic tools in particular for the early detection of tumors is important. Previous studies have compared the gut microbiota composition of stool samples from healthy people versus patient with polyps^27,28^. However, the fecal microbiota is only partially representing the autochthonous microbiota that is in direct contact with the intestinal mucosa^29,30^. Thus, in order to evaluate the role of the indigenous gut microbiota of subjects undergoing colic mucosectomies of polypoid lesions, we performed a pilot study collecting gut biopsies from healthy mucosa and polyps from the same subjects. On these samples, we performed a meta-analysis involving the here reconstructed microbiota composition of adenomatous polyps and publicly available metagenomic datasets of colorectal cancer based on 16S rRNA microbial profiling data^31–33^ in order to search for the occurrence of common microorganisms between polyps and colon cancer. Altogether, these analyses revealed potential novel microbial biomarkers associated with early malignant transformation.

## Materials and Methods

### Patient recruitment within a pilot study

At the Gastroenterology and Digestive Endoscopy Unit, University Hospital of Parma, Italy, 12 patients were enrolled, after obtaining informed consent (Table S1). Exclusion criteria included age >of 90, absence of colic polyps in pancolonoscopy, use of antibiotics within the past 3 weeks, inability to sign informed consent. Colonic Mucosa with Polyp (CMP) and Healthy Marginal Tissue (HMT) were obtained from the same subject collected by endoscopy biopsy forceps, thus generating a total of 24 samples (mean age of subjects: 66 ± 12 years old), immediately frozen at −80 °C and stored until further processing for DNA extraction. DNA was extracted from each sample using the PowerViral^TM^ Environmental RNA/DNA Isolation Kit Sample (Qiagen) following the manufacturer’s instructions (Qiagen Ltd, Strasse, Germany). This study protocol was approved by Ethics Committee of Parma Province and all experiments were performed in accordance with relevant guidelines and regulations. Each patient was characterized by demographic data, level of disease indicators and therapy.

### 16S rRNA gene amplification

Partial 16S rRNA gene sequences were amplified from extracted DNA using primer pair Probio_Uni (5′-CCTACGGGRSGCAGCAG-3′)/Probio_Rev (5′-ATTACCGCGGCTGCT-3′), which targets the V3 region of the 16S rRNA gene sequence^34^. Illumina adapter overhang nucleotide sequences were then added to the partial 16S rRNA gene-specific amplicons, which in turn were further processed by employing the 16S Metagenomic Sequencing Library Preparation Protocol (Part #15044223 Rev. B – Illumina; see also below). Amplifications were carried out using a Verity Thermocycler (Applied Biosystems). The integrity of the PCR amplicons was analysed by electrophoresis on a 2200 TapeStation Instrument (Agilent Technologies, USA).

### MiSeq sequencing of 16S rRNA Gene-based amplicons

PCR products obtained following the amplification of a section of the 16S rRNA gene were purified by a magnetic purification step involving Agencourt AMPure XP DNA purification beads (Beckman Coulter Genomics GmbH, Bernried, Germany) in order to remove primer dimers. The DNA concentration of the amplified sequence library was estimated employing a fluorimetric Qubit quantification system (Life Technologies). Amplicons were diluted to 4 nM and 5 µl of each diluted DNA amplicon sample was mixed to prepare the pooled final library. Paired-end sequencing (250 bp × 2) was performed using an Illumina MiSeq sequencer with MiSeq Reagent Kit v3 chemicals-600 cycles (Illumina Inc., California, USA).

### 16S rRNA-microbial profiling analysis

The fastq files were processed using QIIME^35^ as previously described^34^. Paired-end reads were merged, and quality control implementation allowed the retention of sequences with a length between 140 and 400 bp, mean sequence quality score >25 and with truncation of a sequence at the first base if a low quality sequence within a rolling 10 bp window was found. Sequences with mismatched forward and/or reverse primers were omitted. 16S rRNA Operational Taxonomic Units (OTUs) were defined at ≥97% sequence homology using uclust^36^ and OTUs with less than 10 sequences were filtered. The Biological Observation Matrix (BIOM) obtained was analysed by summarize_taxa.py script in order to get the relative abundance of each taxonomic group for all samples. All reads were classified to the lowest possible taxonomic rank using QIIME^35^ and a reference dataset from the SILVA database^37^. The microbial richness of the samples (alpha-diversity) was evaluated with the Chao1 index through the alpha_rarefaction.py script included in the Qiime software suite using default parameters. Similarities between samples (beta-diversity) were calculated by unweighted uniFrac^35,38^. Hierarchical clusterings were performed with TMeV 4.8.1 (http://www.tm4.org) using Pearson correlation.

### Statistical analyses

SPSS software (www.ibm.com/software/it/analytics/spss/) were used to complete statistical analysis. All data were presented as means ± SD. Furthermore, differential abundance of bacteria taxa and alpha-diversity were tested by one-way analysis of variance (ANOVA).

### Quantitative -PCR

qPCR experiments were based on species-specific primers of *Fusobacterium nucleatum* targeting a previously described prostaglandin transporter-encoding gene^39^. The genome copy-number and the deduced cell number (since the genes targeted were in single copy per genome) was evaluated by comparing the cycle threshold (Ct) values obtained with those from a standard curve. Standard curves were calculated from serial dilutions of a culture with a known cell number (as determined by viable count assessment) for the bacterial strain versus Ct produced for each target gene. The primer sequences were as follows: forward primer 5′-CAACCATTACTTTAACTCTACCATGTTCA-3′ and reverse primer 5′-GTTGACTTTACAGAAGGAGATTATGTAAAAATC-3′. qPCR was performed using the CFX96 system (BioRad, CA, USA). Each PCR reaction mix contained the following: 12.5 μl 2x SYBR SuperMix Green (BioRad, CA, USA), 1 μl of DNA dilution, each of the forward and reverse primers at 0.5 μM and nuclease-free water was added to obtain a final volume of 20 μl. PCR products were detected with SYBR Green fluorescent dye and amplified according to the following protocol: one cycle of 95 °C for 2 minutes, followed by 42 cycles of 95 °C for 5 s and 60 °C for 30 s. Melting curve: 65 °C to 95 °C with increments of 0.5 °C/s. In each run, negative controls for each primer set were included. Cycle thresholding was calculated using the automated settings for Biorad CFX Manager 3.1 software (BioRad). The entire qPCR experiment was performed a second time using the same samples and methods as outlined above, for the purpose of replication, and very similar results were obtained.

### Data Deposition

The 16S rRNA profiling data sequenced in this study were deposited in SRA database under accession number PRJNA415554.

## Results and Discussion

### Patients and pathological data

A total of twelve patients with polyps were included in this pilot study. From those, histological analyses revealed that four corresponded to adenomatous polyps and eight to hyperplastic polyps. All adenomatous polyps were found in males, while the remaining hyperplastic polyps were collected from three males and five females. Two biopsies were collected from each patient, one corresponding to the polyp and one to healthy mucosa. Patient characteristics and details of endoscopic treatment performed are outlined in Table S1.

### Cataloguing of polyp-associated microbiota and comparison to healthy mucosa microbiota

Twelve Colonic Mucosa with Polyp (CMP) and corresponding Healthy Marginal Tissue (HMT) samples (i.e. total of 24 samples), retrieved from twelve hospitalized subjects, were used to evaluate the microbiota taxonomic profile by means of 16S rRNA gene sequencing analysis as described previously^34^.

Next generation sequencing of these samples produced a total of 2,059,497 sequencing reads with an average of 85,812 reads per sample (Table S2). Subsequent quality and chimera filtering produced a total of 1,479,134 filtered reads with an average of 61,631 reads, and ranging from 33,783 to 202,018 reads per sample (Table S2).

Rating of rarefaction curves based on the Chao1 biodiversity indexes calculated for the two subgroups of sequenced read pools showed that both curves tend to reach a plateau confirming the high accuracy of the performed 16S rRNA profiling analysis. Furthermore, average rarefaction curves revealed a different trend of bacterial richness of the gut microbiota of healthy samples compared to that found in the polyp sample group, i.e. higher level of HMT vs CMP samples (Fig. S1). However, these analyses displayed no statistical significance, since the ANOVA calculated at the highest rarefaction depths reached by all the samples, i.e. Chao1 average is 1895 ± 480 in HMT and 1678 ± 634 in CMP indicated a p-value > 0.05 (Fig. S1).

### 16S rRNA profiling of HMT and CMP samples

Inspection of predicted taxonomic profiles at phylum level for all samples showed that Firmicutes represented the dominant phylum (average of 57.59% ± 10.48%) of the colonic mucosa samples, out-numbering the Bacteroidetes (average of 9.24% ± 8.01%) and Proteobacteria (average of 10.26% ± 8.19%) phyla.

**Figure 1:**
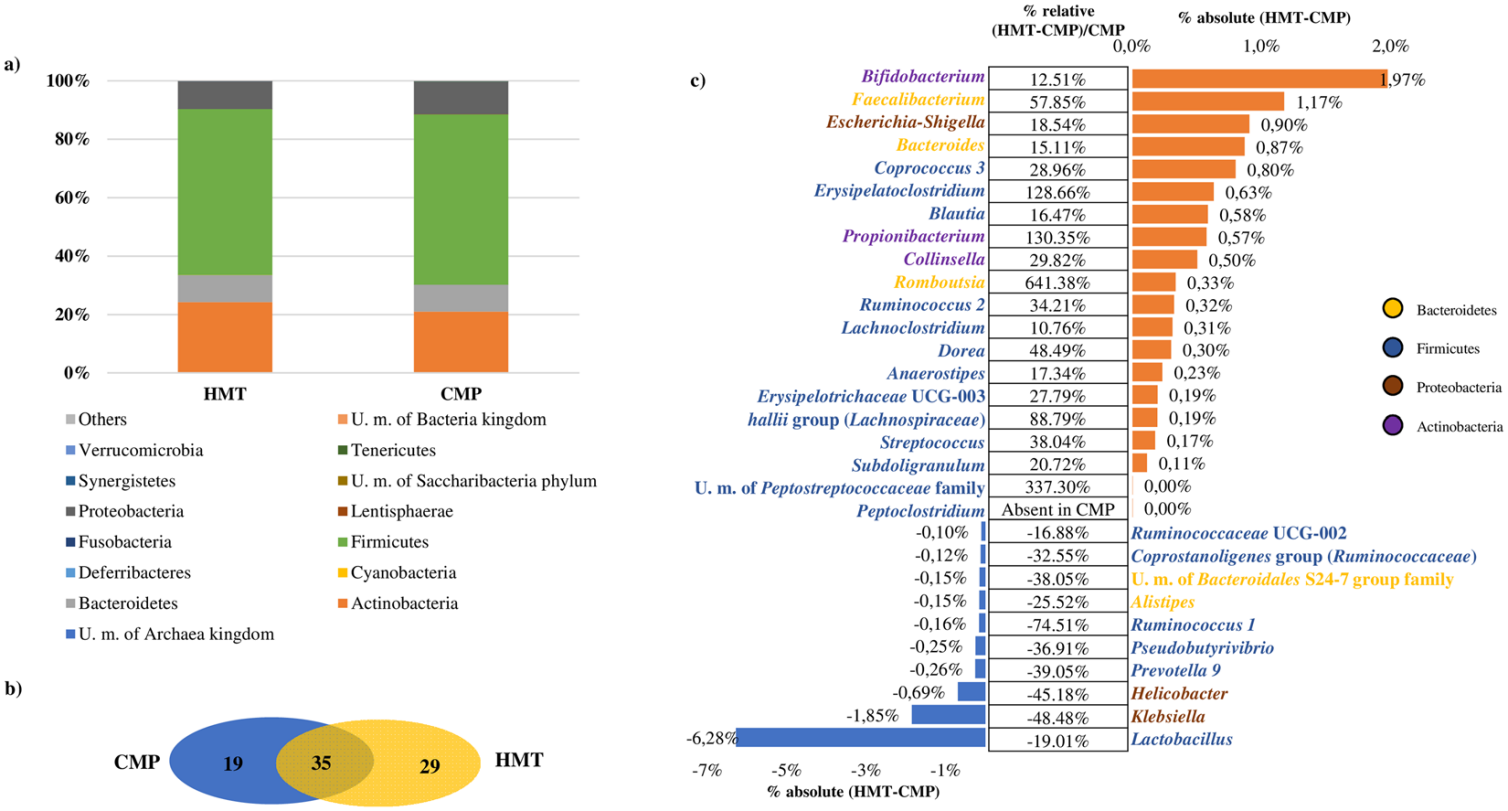
Exploration of the diversity in HMT and CMP groups. Panel a represents a bar plot of the bacterial phyla identified in the 24 analyzed samples. Panel b depicts a Venn diagram illustrating the total, unique and shared number of OTUs predicted for HMT and CMP datasets. Panel c shows the variation in terms of relative abundance of >0.4%, and showing either an increase of >10% or decrease of <−16% in CMP data sets as compared to those obtained from HMT samples.

Nevertheless, no major shifts were observed in bacterial populations between healthy mucosa and Polyp-associated mucosa, although we did observe a moderate reduction of Actinobacteria (relative percentage difference of −13.43%) and Bacteroides (relative percentage difference of −1.02%) in CMP and a concomitant modest increase of Firmicutes (relative percentage difference of 2.84%) compared to HMT (Fig. 1a–c).

Furthermore, at genus level, we identified 308 genera of which 225 appear to be present in at least one sample of HMT and CMP groups. In detail, 35 bacterial taxa were present in all 24 samples, thus representing the mucosa core microbiota, i.e. the permanent and common members of the microbial community^40^, which may include specific species important for the maintenance of an efficiently functioning gut ecosystem^41^ (Fig. 1b).

Moreover, HMT displayed 29 unique taxa representing the 0.015% of the total genera abundance, while CMP showed 19 unique representing 0.008% of the total genera abundance (Fig. 1b).

### Taxa associated with HMT and CMP

In order to further identify differences in microbiota composition between CMP and HMT patients, we focused on 30 taxa, which were shown to be present as the absolute change of >±0.1% or that displayed a significant variation in terms of relative abundance (p-value < 0.05). In this context, these 30 taxa varied in terms of relative abundance, encompassing an increase ranging from 10% (*Lachnoclostridium*) to 641% (*Romboutsia*), or a decrease ranging from −16% (*Ruminococcaceae* UCG-002) to −74% (*Ruminococcus* I) in HMT datasets compared to those obtained from CMP samples (Fig. 1c). Interestingly, members of the Actinobacteria phylum such as the *Bifidobacterium* genus are present in higher abundance in HMT as compared to CMP samples. Bifidobacteria species are known to represent typical gut microorganisms with presumed positive effects on human health and recently their absence has been linked to various disease conditions^42–45^. In addition, the *Faecalibacterium* genus, which is among bacteria with purported health-promoting activities^46^, as well as *Bacteroides* taxa, which have been found at a lower abundance in IBD patients^47^ are present at a higher level in HMT samples. Moreover, *Romboutsia* taxa is the genus with the highest relative abundance in HMT vs. CMP samples. Notably, *Romboutsia* is a recently described bacterial genus^48,49^ commonly identified in the human gut^50,51^, and often associated with a healthy status of patients^50,51^. The drastic reduction of members of this particular genus (−86,51%) in mucosa associated with polyps may represent a potential microbial indicator of a disease condition^50^.

In contrast, the higher presence of *Helicobacter* and *Klebsiella*, which are typically associated with carcinogenesis^52,53^ in CMP samples reflect a typical disease condition. Interestingly, among taxa showing a significant variation in terms of relative abundance (p-value < 0.05), Unclassified member (U. m.) of *Peptostreptococcaceae* family occurs with an increased relative abundance of 337.30% in HMT vs. CMP samples, while *Peptoclostridium* is totally absent in all analyzed CMP samples.

In order to integrate and confirm the observed differences in relative abundance between HMT and CMP samples, we defined the trend (increase or decrease) for each bacterial taxa in healthy subjects versus polyp condition. Considering all patiens, we identified that only the genus *Romboutsia* displayed the same trend in at least 80% of HMT samples, while all taxa trend of CMP were <75%. These results reinforce the notion that *Romboutsia* may play a key role in maintaining the health status of the host and render this taxon a very valuable candidate as biomarker of intestinal dysbiosis.

### Identification of possible cancer biomarkers

In order to reinforce our taxonomical observation and to enlarge the CMP database, we performed a survey and selection of 16S rRNA-based polyp-associated metagenomic datasets deposited in the publicly NCBI database. However, the published polyp-associated metagenomic datasets are based on fecal samples^27,28^. In this context, it is worth mentioning that previous studies on healthy patients or children with rectal bleeding indicated that fecal and mucosal samples belonging to the same individual displayed a very dissimilar microbial composition^29,30^. It has been reported that, based on different statistical analyses, fecal and mucosal samples cluster separately, at family and species taxonomic level^29,30^. To circumvent the issue of absence of data from polyp-associated biopsies, we decided to use human CRC datasets. In fact, as discussed above, CRC has been considered to represent a later stage of adenomas^2,54^. Consequently, we retrieved 293 samples from the four publically available CRC BioProjects^31–33^ (Table S3). In detail, we selected the datasets based on Illumina or 454 technologies including both healthy and CRC samples obtained from biopsies collected from the human large intestine (Table S3). Notably, to avoid biases caused by different bioinformatic analyses pipelines^55^, the sequence reads pools of each study were analyzed involving the same custom script^56^ based on the QIIME software suite^35^ used here for the 24 HMT/CMP samples. These datasets were enriched with the CMP and HMT 16S rRNA microbial profiling data obtained and described in our current study.

**Figure 2:**
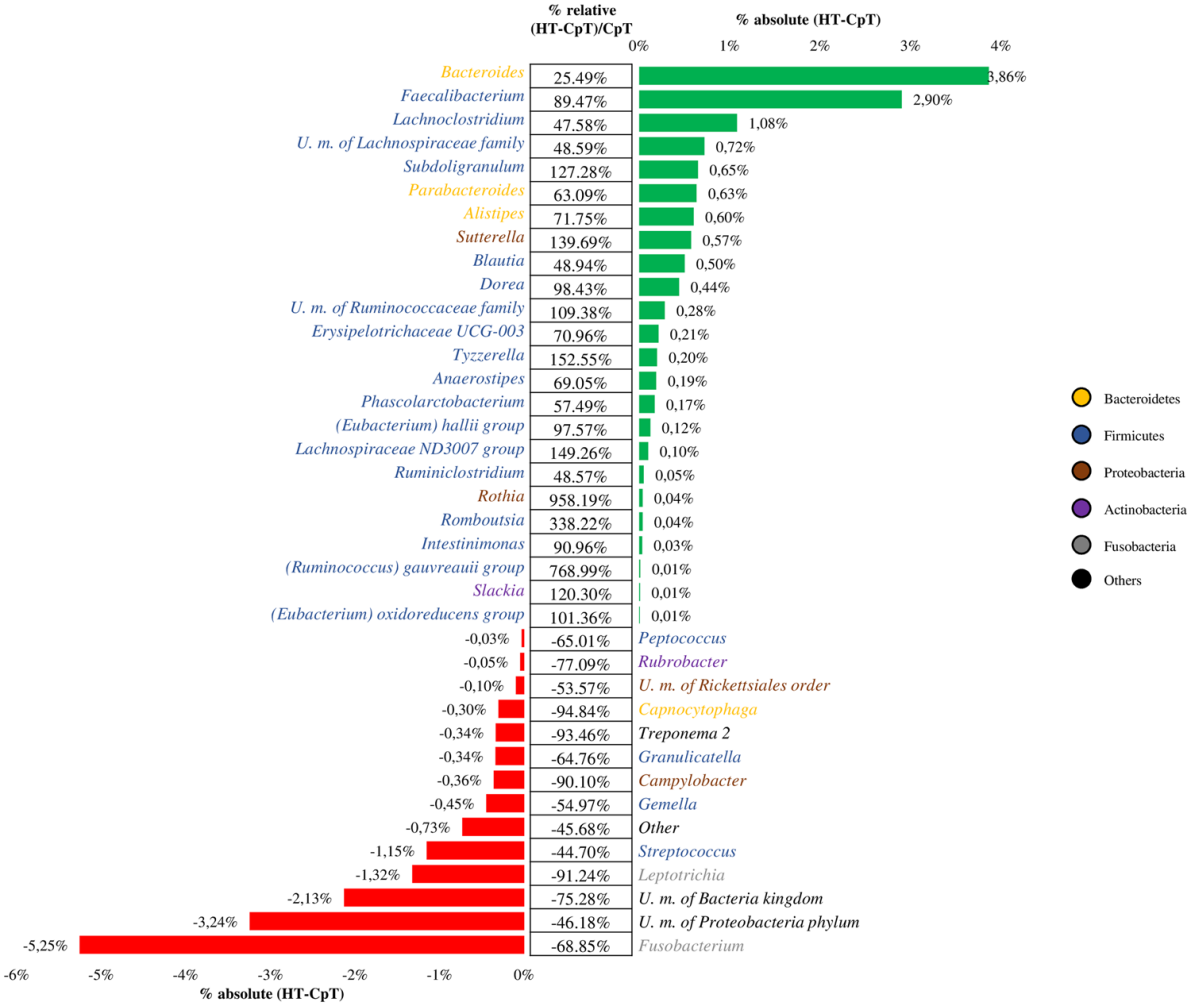
Exploration of the diversity in HT and CpT groups. The image shows the variation in terms of relative abundance of HT and CpT samples. Only taxa with a p-vale <0.05 were shown.

Evaluation of the difference between all Healthy Tissue (HT) and CRC-polyps Tissue (CpT) samples highlighted 38 genera with a p-value < 0.05 calculated thought Analysis of variance (ANOVA) (Fig. 2). Furthermore, we investigated the difference between each case-control, HT vs. CRC and HMT vs. CMP, in order to calculate the trend percentage.

**Figure 3:**
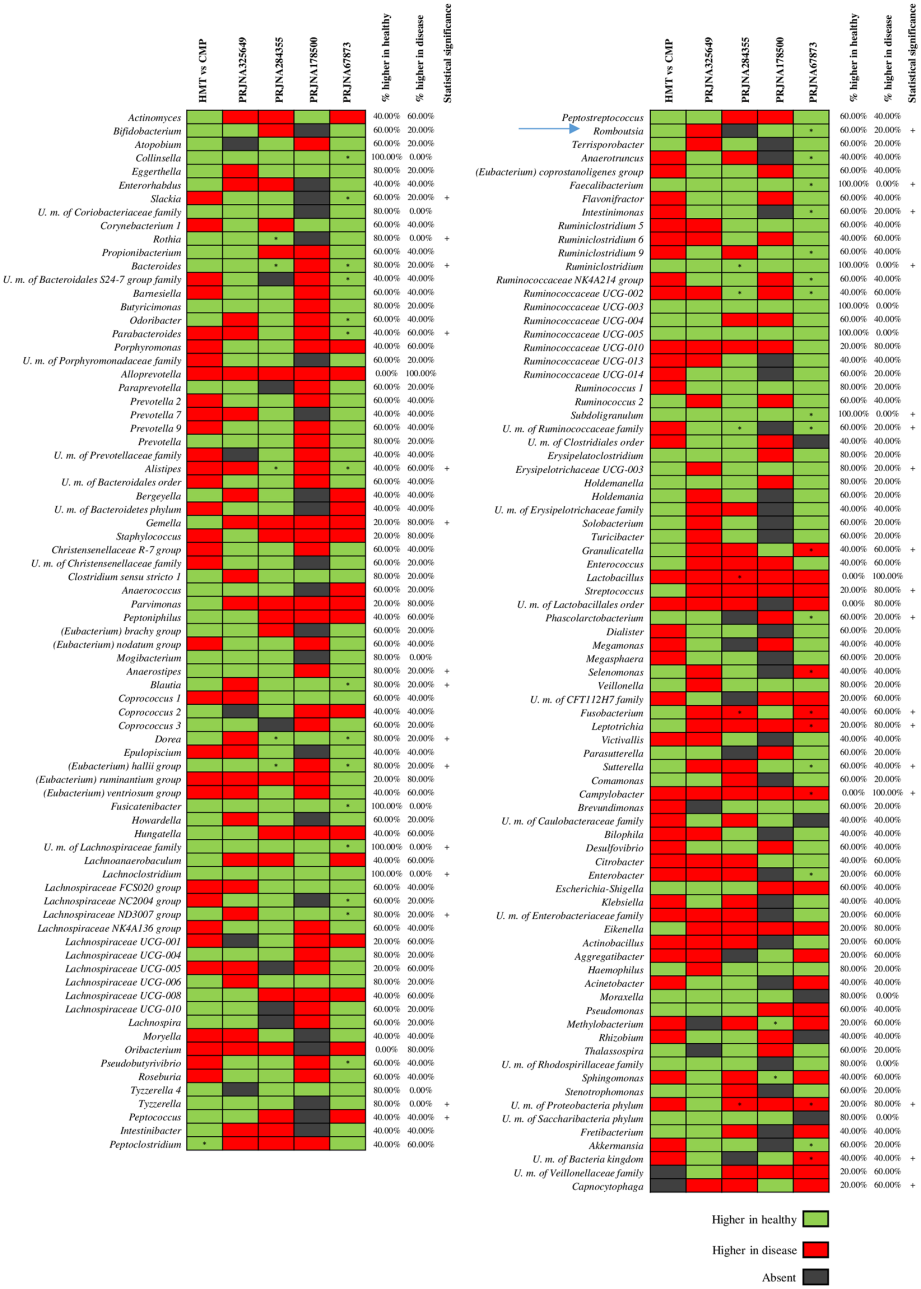
Evaluation of taxa trend in the gut microbiota of HT and CpT samples. Asterisks indicate statistically significant differences between each case-control for all five datasets (P-value <0.05). The heatmap shows the taxonomic trend of each 16S rRNA gene-based metagenomic dataset. Names of identified bacterial taxa are listed on the left, while names of sample groups used are listed at the top. Healthy Marginal Tissue (HMT); Colonic Mucosa with Polyps(CMP); ColoRectal Cancer (CRC); all Healthy Tissue (HT) and CRC-polyps Tissue (CpT). The green color represents high abundance in healthy cases, red color represents high abundance in disease cases and black color represents the absence of the bacterial taxa. Columns show the studies analyzed and rows indicate the bacterial taxonomy at genus level.

Association of ANOVA and trend percentage analyses indicated that CpT samples had a higher abundance of genera belonging to Proteobacteria and Fusobacteria phyla, specifically *Campylobacter* (taxonomic trend prevalence of 100% in CpT) and *Fusobacterium* (taxonomic trend prevalence of 60% in CpT) compared to healthy samples (Fig. 3). Interestingly, these taxa are commonly found in patients with CRC^57^, with the *Fusobacterium* genus frequently being associated with tumor progression^58^. Moreover, CpT samples possessed microbial genera commonly involved in development of intestinal disease or pathologies, such as *Streptococcus* (taxonomic trend prevalence of 80.00% in CpT) and *Gemella* (taxonomic trend prevalence of 80% in CpT)^59,60^. In contrast, HT samples showed higher abundance of ‘beneficial’ bacterial genera, as well as U. m. of *Lachnospiraceae* family and *Faecalibacterium* (taxonomic trend prevalence of 100% in HT), *Bacteroides* (taxonomic trend prevalence of 80% in HT), and *Romboutsia* (taxonomic trend prevalence of 60% in HT) compared to CpT.

Furthermore, focusing on genera with a taxonomic trend with a prevalence of 100% and a significant p-value (<0.05) calculated between HT and CpT (Fig. 3), it was possible to identify five taxa characteristic of HT and one taxon of CpT subjects. In detail, healthy samples showed high abundance of U. m. of *Lachnospiraceae* family, *Lachnoclostridium*, *Faecalibacterium*, *Ruminiclostridium* and *Subdoligranulum*, while disease-associated CpT samples showed high abundance of *Campylobacter*. These results reflect the assumption that genera belonging to *Lachnospiraceae* and *Ruminococcaceae* families may protect healthy subjects from intestinal disease^61^, such as through the production of short-chain fatty acids (SCFAs)^62^. In contrast, the higher abundance of bacteria belonging to *Campylobacter* genus may confirm its correlation with the development of gastrointestinal diseases^63,64^.

Overall, we found a correspondence between the trend that we retrieved in our HMT vs CMP samples, and that of the HT vs CRC samples, revealing a small number of microbial taxa that could be considered as bacterial biomarkers for early detection of tumor or, conversely, reflecting a healthy condition.

### Quantification of *Fusobacterium nucleatum* by qPCR

**Figure 4:**
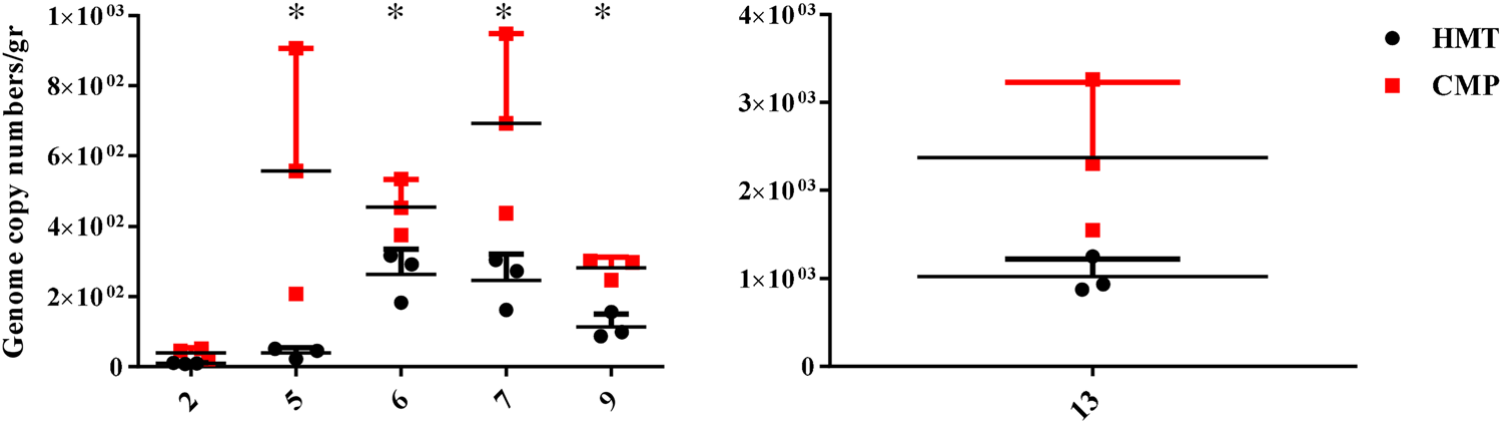
Quantification of *Fusobacterium nucleatum* by qPCR. Results of qPCR are represented in a grouped graph where each sample is symbolized by an individual value plot. The *y*-axis represents the genome copy number/gr of *Fusobacterium nucleatum* and *x*-axis shows the patient number. For each patient, HTM biopsies are schematically represented by a black circle and relative CMP portion in red square. Statistical differences were calculated by t-test (*P < 0.04).

Since several studies associated the presence of *Fusobacterium nucleatum* with a formation and progression of colorectal cancer^39,64–67^ the relative abundances of this species were measured by qPCR in all 24 samples (HTM and CMP biopsies of 12 patients). Notably qPCR analyses displayed that this bacterium has been found in six patients (i.e., 2, 5, 6, 7, 9 and 13) with a genome copy number ranging from 2.24E1 to 1.25E3 for the HTM samples, and a genome copy number range of 2.08E2 to 3.26E3 for CMP biopsies (Fig. 4). These data clearly indicated that the total load of *Fusobacterium nucleatum* was significantly (p-value < 0.04) higher for samples 5, 6, 7 and 9 in the CMP vs. HMT mucosa for each patient. Interestingly, sample 13, showed a total load of *Fusobacterium nucleatum* higher respect all other samples, i.e., a mean value of 1.02E3 for the HMT as compared to a mean value of 2.37E3 for the CMP (p-value < 0.04) (Fig. 4). These preliminary data suggest that this species can, as previously indicated, not only be considered an important bacterial biomarker for the diagnosis of colorectal cancer^39,66^, but may also serve as a putative valuable microbial biomarker for early diagnosis of malignant displacement of polyps.

## Conclusions

Recent findings have shown that intestinal diseases are correlated with microbiota alterations such as in the case of ulcerative colitis^45,68^.

The contribution of gastrointestinal microbiota in the progression and development of colorectal cancer is complex and not fully understood^67^, but previous *in vivo* murine based-analyses highlight the potential role of many individual bacteria to promote carcinogenesis^28,69^.

In the current pilot study, we compared bacterial population in mucosa samples of patients with polyps or CRC, versus healthy mucosa, showing that the *Romboutsia* genus is more abundant in healthy compared to polyp-associated tissue. Remarkably, the relative abundance of *Romboutsia* in CRC tissue was found to be drastically reduced. Thus, we may argue that the absence of this microbial genus is a first indicator of an alteration of the mucosa. Remarkably, this microbial taxon has already been observed for other gut-associated diseases^49–51^.

Interestingly, the *Peptoclostridium* genus was undetectable in all CMP sample tested, while analysis of the HMT samples revealed a relatively high number of U. m. of *Peptostreptococcaceae* family.

Our findings were in line with the findings of a meta-analysis in which we compared the CMP samples with CRC samples from publically available metagenomic datasets^31–33^. Our metagenomic analysis revealed that CpT samples had a higher abundance of the Fusobacteria phylum compared to healthy samples. Interestingly, the *Fusobacterium* genus is often involved in tumor progression^58,70^.

At genus level, we identified five taxa characteristic of HT and one taxon typical of CpT subjects. In detail, healthy samples were shown to contain a high abundance of U. m. of *Lachnospiraceae* family, *Lachnoclostridium*, *Faecalibacterium*, *Ruminiclostridium* and *Subdoligranulum*, while CpT samples showed high abundance of *Campylobacter*. The higher abundance of bacteria belonging to the *Campylobacter* genus in CRC mucosa, as well as polyp-associated samples reinforces its correlation with the development of intestinal pathologies^63,64^. On the other hand, genera belonging to *Lachnospiraceae* and *Ruminococcaceae* families appear to be correlated to beneficial effects on the host as described previously^61^.

Gut microbiota include a very complex array of microorganisms and its compositional changes have been shown to impact on human health status^71,72^. The identification of microbial players that are distinctly altered in their abundance in CRC-associated samples may allow early diagnosis of malignant transformation as well as the identification of protective bacteria missing in polyps or CRC tissue.

In conclusion, the data obtained in this pilot study have allowed us to identify possible microbial biomarkers associated with polyps and/or CRC. However, such findings need further validation by analysis of a larger number of samples, in order to further substantiate and fully characterize such potential microbial biomarkers of CRC development, as well as beneficial bacteria, and to identify their functions and interactions with the host.

## Electronic supplementary material

Supplementary files

Table S3
