# Supplementary material for: Mucosal microbiota of intestinal polyps reveals putative biomarkers of colorectal cancer
Source: Sci Rep. 2018 Sep 18;8:13974. doi: 10.1038/s41598-018-32413-2 (PMC6143603; doi:10.1038/s41598-018-32413-2)
Supplement: Supplementary file 1 — Supplementary files [file 41598_2018_32413_MOESM1_ESM.pdf]

Mucosal microbiota of intestinal polyps reveals putative biomarkers of colorectal cancer

Marta Mangifesta[1], Leonardo Mancabelli[1], Christian Milani[1], Federica Gaiani[2], Nicola de' Angelis[3], Gian Luigi de'Angelis[2], Douwe van Sinderen[4], Marco Ventura[1,5] and Francesca Turroni[1,5]

Laboratory of Probiogenomics, Department of Chemistry, Life Sciences and Environmental Sustainability, University of Parma, Parma, Italy[1]; Gastroenterology and Endoscopy Unit, University Hospital of Parma, Parma, Italy[2]; Department of HPB Surgery and Liver Transplantation, Henri-Mondor Hospital, Université Paris Est-UPEC, Créteil, France[3]; School of Microbiology & APC Microbiome Institute, University College Cork, Cork, Ireland[4]; Microbiome Research Hub, University of Parma, Parma, Italy[5];

Supplementary information

Additional files

Table S1: Clinicopathologic data of 12 patients with polyps after colonoscopy

Table S2: 16S rRNA microbial profiling data

Table S3: List of the public CRC datasets

Figure S1: Evaluation of alpha-diversity in CMP and HMT samples

Table S1. Clinicopathologic data of 12 patients with polyps after colonoscopy.

Samples	Sex	height (cm)	weight (kg)	BMI	symptoms	bowel motion	n° polyps	CMP sites	HMT sites	histology
**1**	M	180	96	29.6	pain/abdominal swelling	infrequent	2	rectum	sigmoid	hyperplastic
**2**	F	165	60	22.2	pain/abdominal swelling	infrequent	1	sigmoid	rectum	hyperplastic
**3**	F	180	75	23.1	pain/abdominal swelling	regular	2	sigmoid	rectum	hyperplastic
**4**	M	175	60	19.6	no symptoms	regular	5	transverse colon	sigmoid	hyperplastic
**5**	M	160	68	26.5	no symptoms	regular	2	sigmoid	sigmoid	tubular adenomas
**6**	M	175	83	25.6	pain/abdominal swelling	regular	2	transverse colon	sigmoid	tubular adenomas
**7**	M	162	71	26.1	no symptoms	regular	2	ascending colon	sigmoid	tubular adenomas
**8**	F	145	37	17.6	hematochezia	constipation	1	rectum	sigmoid	hyperplastic
**9**	F	165	72	26.5	pain/abdominal swelling	infrequent	1	sigmoid	rectum	hyperplastic
**11**	F	153	72	30.7	no symptoms	regular	2	sigmoid	rectum	hyperplastic
**12**	M	176	84	27.2	no symptoms	regular	1	sigmoid	sigmoid	hyperplastic
**13**	M	162	80	30.5	no symptoms	regular	1	ascending colon	sigmoid	tubular adenomas

Table S2: 16S rRNA microbial profiling data.

Samples	Number of sequenced pe reads	Number of pe reads with mean quality > 20	Number of merged pe reads	Human sequences	Length outside bounds of 100 and 400	Ambiguous bases	Homopolymers > 7	Mismatch in primers >1	Reverse primer not found	Final Read Number
**1_HMP**	76352	75090	66727	556	0	0	3	31987	68	34113
**1_CMP**	99597	96947	89782	7454	0	0	4	28562	103	53659
**2_HMP**	262569	249759	242886	34817	0	0	17	5835	199	202018
**2_CMP**	120637	117531	114142	3698	0	0	2	2869	72	107501
**3_HMP**	70571	68276	60599	12564	0	772	29	3025	81	44127
**3_CMP**	62914	59825	53078	16100	0	578	31	2535	51	33783
**4_HMP**	65358	64641	60572	3578	0	1051	24	4918	115	63516
**4_CMP**	41247	39954	35490	15046	0	738	7	4171	102	59414
**5_HMP**	91031	89145	82349	12725	0	1239	1	3872	109	59684
**5_CMP**	78916	77830	71003	6571	0	808	3	3050	82	39719
**6_HMP**	77288	76378	70066	5161	0	1684	8	4183	135	70286
**6_CMP**	59515	58448	51852	8190	0	1411	1	4043	101	60432
**7_HMP**	95790	94194	84655	8359	0	884	9	3838	144	51676
**7_CMP**	74620	73970	66399	410	0	710	16	3179	68	42553
**8_HMP**	78125	76939	70644	14093	0	825	1	4052	180	52284
**8_CMP**	101175	98829	91152	44626	0	558	14	3538	99	49062
**9_HMP**	64850	64070	57440	98	0	825	1	4052	180	52284
**9_CMP**	76965	75690	68954	15683	0	558	14	3538	99	49062
**11_HMP**	84825	83923	76741	3315	0	1538	2	4067	114	67705
**11_CMP**	66890	65438	57745	1064	0	1228	1	3651	98	51703
**12_HMP**	87497	85409	78754	9015	0	1307	6	4065	86	64275
**12_CMP**	77867	75333	68322	1779	0	950	4	4755	81	60753
**13_HMP**	80426	79034	73216	5866	0	835	9	4447	107	61952
**13_CMP**	64472	63420	56664	4241	0	0	2	4511	337	47573

Supplementary Figure legend

Figure S1. Evaluation of alpha-diversity in CMP and HMT samples. Panel A shows the average rarefaction curve representing variation of the Chao1 diversity index at increasing sequencing depth of CMP and HMT samples. Panel B displays the average rarefaction curve representing all 24 samples: dashed lines represents all 12 CMP samples and straight lines represents all 12 HMT samples. X-axis show the number of reads.

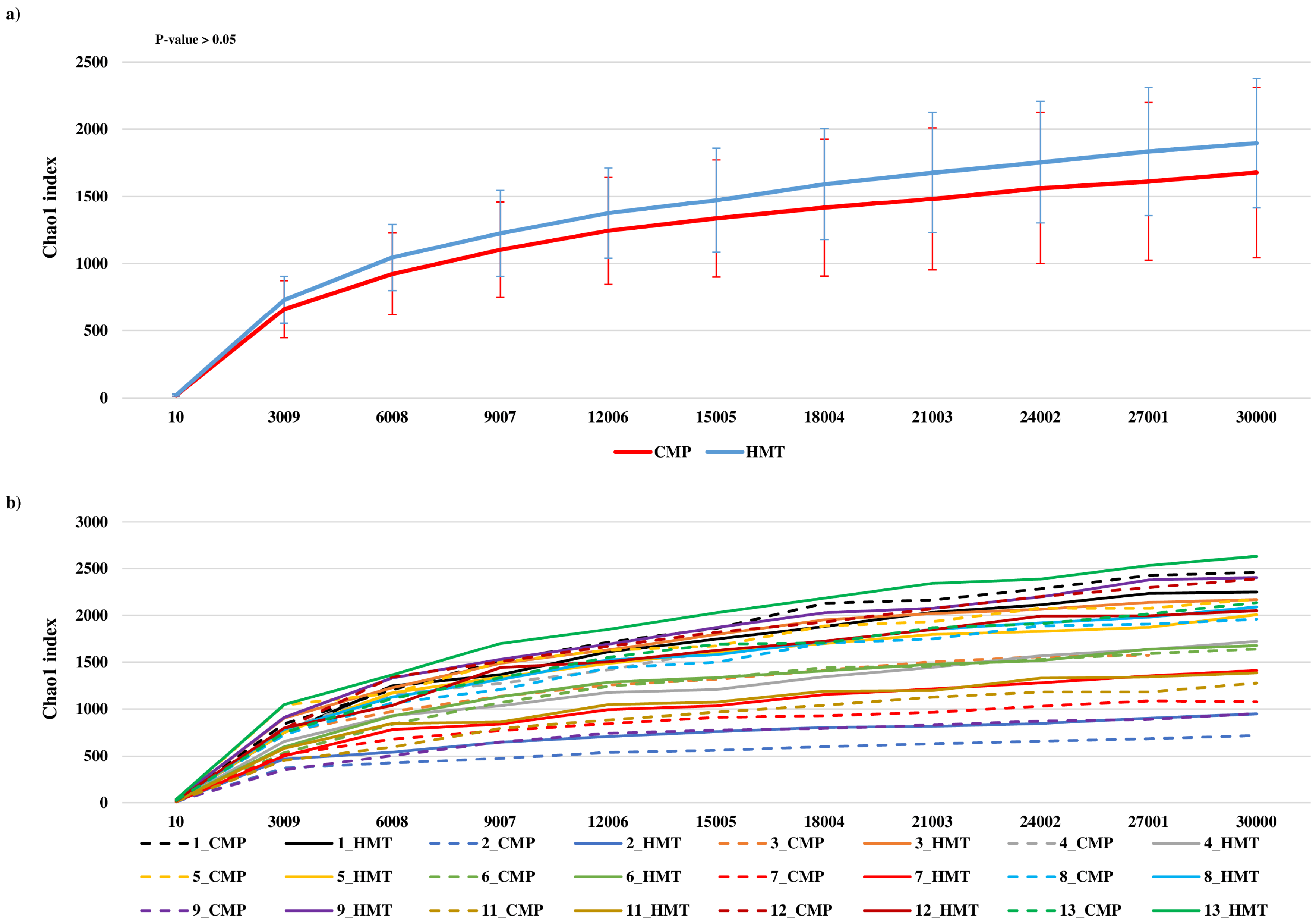
a)
P-value > 0.05
Chao1 index
2500
2000
1500
1000
500
0
10
3009
6008
9007
12006
15005
18004
21003
24002
27001
30000
CMP
HMT
b)
Chao1 index
3000
2500
2000
1500
1000
500
0
10
3009
6008
9007
12006
15005
18004
21003
24002
27001
30000
1_CMP
1_HMT
2_CMP
2_HMT
3_CMP
3_HMT
4_CMP
4_HMT
5_CMP
5_HMT
6_CMP
6_HMT
7_CMP
7_HMT
8_CMP
8_HMT
9_CMP
9_HMT
11_CMP
11_HMT
12_CMP
12_HMT
13_CMP
13_HMT

Figure S1
